# Dural and Leptomeningeal Spine Metastases of Breast Cancer

**DOI:** 10.1155/2019/4289362

**Published:** 2019-05-29

**Authors:** Fernando Matos, Luís Cerqueira

**Affiliations:** ^1^Radiology Unit, Centro Hospitalar Tondela-Viseu, EPE, Portugal; ^2^Neuroradiology Unit, Centro Hospitalar Universitário Lisboa Central, EPE, Portugal

## Abstract

We present a case of a 57-year-old female diagnosed with invasive ductal breast cancer, which was treated and in remission for 12 years. In 2018 she presented a progressive dorsal back pain, which prevented her from performing basic tasks. An MR study was performed and revealed the presence of an extramedullary metastatic sleeve located in the thoracic intradural space. Concomitant multiple small nodular foci were adhering diffusely to the spinal cord, compatible with leptomeningeal metastatic disease. The occurrence of both forms of spread in the spine is uncommon, and its distinction on imaging is of particular importance taking into account the differences in treatment approach and prognosis.

## 1. Introduction

With the advent of improved systemic therapies, there has been an increase in the number of long-term survivors of cancer disease, which is particularly relevant in the case of breast cancer. Despite this improvement in survival, there was also an increase in the incidence of late onset metastases, which constitutes a concerning clinical issue and a challenge for current oncology [1].

Intradural neoplasms are located outside the spinal cord but within the dural sheath. The growth of these masses can cause spinal cord compression related symptoms, thus representing a significant cause of morbidity.

The leptomeninges, on the other hand, are a rare location for metastases [2], occurring more frequently in patients with progressive lung and breast cancer. Its presence implies a poor prognosis and is considered virtually a terminal manifestation in the central nervous system [3]. Nevertheless, cases have been described in which this was the first manifestation of the disease.

## 2. Case Presentation

We present a case of a 44-year-old female patient diagnosed in 2006 with a bifocal invasive ductal breast cancer, HER-2 positive, who underwent mastectomy, chemotherapy, and radiotherapy. The patient was in remission and was under tamoxifen for five years. Twelve years after the initial diagnosis, she developed debilitating dorsal pain, and an MR of the spine was performed.

**Figure 1:**
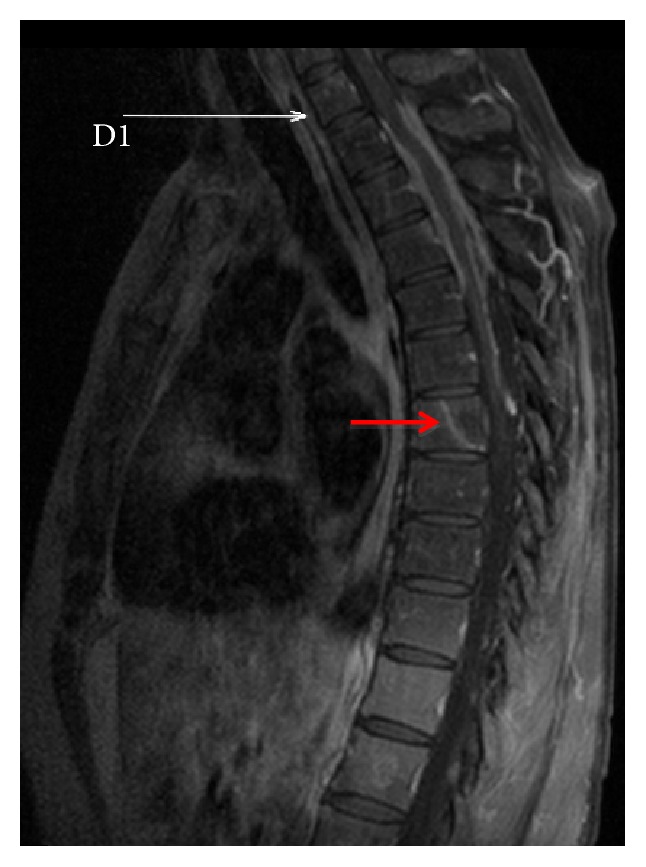
Sagittal gadolinium-enhanced T1-weighted Turbo Spin Echo Fat Sat MR image. The dorsal spinal cord is deformed and compressed by a sleeve of enhancing tissue, centered at the T2-T3 levels and extending between T2 and T7. The posterior body of T7 is infiltrated by a blastic lesion (hypointense on T1, T2, and STIR sequences) with peripheral enhancement (red arrow) highly suggestive of a bone metastasis.

**Figure 2:**
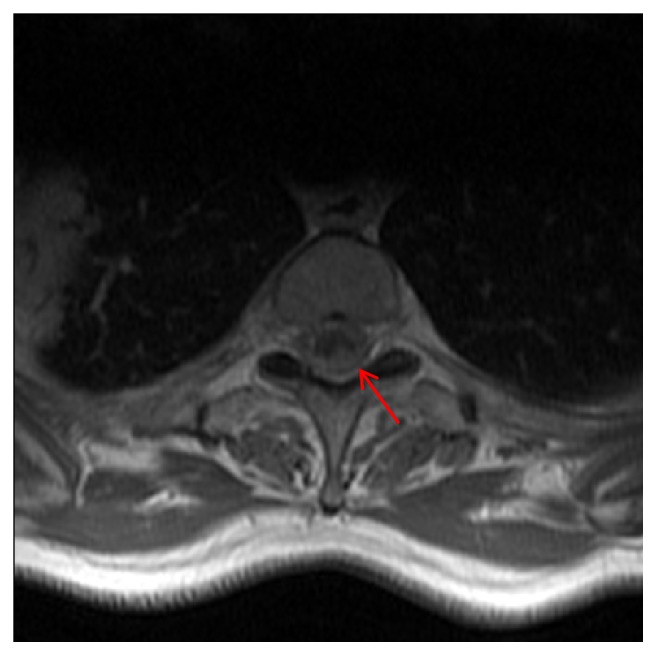
Axial gadolinium-enhanced T1-weighted Turbo Spin Echo MR image. An enhancing mass surrounds, deforms, and compresses the spinal cord all around its perimeter but most exuberantly in the left posterior side (red arrow).

**Figure 3:**
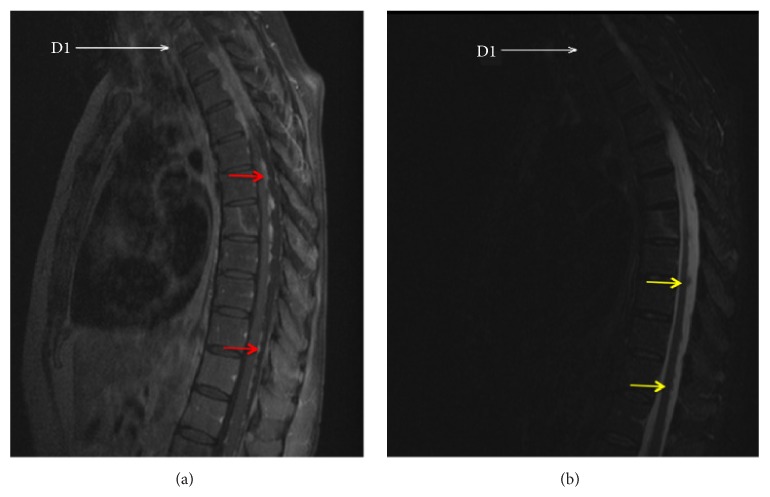
(a) Sagittal gadolinium-enhanced T1-weighted Turbo Spin Echo Fat Sat MR image. Multiple round enhancing foci (red arrows) sticking to the posterior medulla, corresponding to diffuse leptomeningeal spread of metastases. (b) Sagittal T2 Short Time Inversion Recovery (STIR) MR image. The leptomeningeal metastases are also well depicted as hipointense nodules on the STIR sequence (yellow arrows) surrounded by the T2-hyperintense CSF.

The study revealed signs of diffuse medullary metastatic disease, assuming a sleeve-like appearance in the dorsal segment (Figure 1), filling the perimedullary subdural space, most significantly at the T2-T3 level, and molding the posterior medulla (Figures 1 and 2). Additionally, leptomeningeal metastatic spread was also observed as disperse small nodular foci, sticking to the spinal cord and the dorsal roots (Figures 3(a) and 3(b)). There were no signs of intramedullary metastatic spread or signs of cerebrospinal fluid (CSF) blockage. Both the leptomeningeal and the dural components of the disease depicted homogenous uptake of gadolinium. Besides the above-described lesions, there was evidence of bone deposits in the right pedicle of T4 and the vertebral body of T7 (Figure 1). No signs of brain or intramedullary metastatic disease were found.

Given the described findings and symptoms, radiotherapy (RT) treatment (30Gy) was delivered, being successful in the reduction of the volume of the metastatic sleeve at the thoracic level. As a consequence there was a partial improvement in the symptoms and reduction of the analgesic drug dose for eight months now. The patient is currently under surveillance and is also being consulted in a pain management unit. Hormonotherapy, consisting of anastrozole, an aromatase inhibitor, was also prescribed.

## 3. Discussion

Central nervous system (CNS) metastasis occurs quite frequently in cases of primary breast cancer. In the CNS, the cerebral parenchyma stands out as being the most frequent localization of metastatic disease. The average life expectancy in these patients is reduced to 9 months on average, with an estimated one-year survival of 20%. [2]

The long period between the initial diagnosis and the appearance of the dural and leptomeningeal disease makes this case an uncommon occurrence. According to literature, the median time for the onset of leptomeningeal spread is 18 months [3]. In the present case, there is a 144 month interval between the primary diagnosis and the CNS involvement.

Literature tends to separate leptomeningeal from the dural metastases incidence. The true incidence of occurrence of both at the same time is challenging to define and the published articles that address the issue are scarce. A study, by Rumana et al., [4] focused on the involvement of the dura by brain metastasis. Of a total of 425 patients with brain metastatic disease, 22 had dural involvement by metastasis and only 3 (0,7%) had concurrent leptomeningeal and dural metastases. The limitation is that the study focused essentially on the brain and not the spine. However, the similarities of the mechanisms of spread could lead us to assume that the percentages in the spine would not be very different.

Studies have shown that spinal dural and leptomeningeal spread should be regarded as different forms of disease, each carrying a different prognosis [5]. The mechanisms of the emergence of dural metastases include hematogenous spread and surgical seeding [6]. Dural involvement from metastasis may also result from adjacent metastatic involvement of the skull, brain [6], or the spine, the latter corresponding to the most likely way of the spread in this case. When it comes to leptomeningeal disease, several studies suggested that mechanical spread of tumor cells to the CSF space could contribute to its development [7] which is probably the case since the highest tumor load was located in the thoracic level.

In breast cancer, leptomeningeal spread occurs most often with triple negative and lobular tumor subtypes [8]. In this case, the tumor depicted HER-2 positive receptors, an uncommon feature for leptomeningeal spread, notwithstanding the known increase in the incidence of brain parenchymal metastases of this tumor subtype [9]. Patients with dural metastases may carry a better prognosis than those displaying leptomeningeal spread [5].

Treatment may differ for each of the entities: in the case of dural metastasis, surgery may be indicated in selected cases. Radiation therapy, in the case of spinal cord compression and/or nonresectable disease, should be regarded as a valid option [5, 10]. In the case of leptomeningeal metastases, intrathecal or systemic chemotherapy (e.g., capecitabine) is considered an adequate form of treatment [5, 11], although CSF concentrations of the drug in the case of systemic chemotherapy may still be an issue [12]. Craniospinal axial irradiation of the whole neuroaxis could be an option to treat leptomeningeal disease, but its associated toxicity (myelosuppression and enteritis) is regarded as being too harmful for routine use [11].

Although CSF culture makes the definite diagnosis of leptomeningeal spread, this was not performed in this particular case given the very suggestive findings on MR. According to literature, an abnormal MR study is sufficient to reach the diagnosis of leptomeningeal metastases when the clinical context suggests that possibility even if CSF cytological results are negative. [13]. Also noteworthy is the fact the lumbar puncture is an invasive procedure and the sensitivity of the first puncture that only reaches 50 to 60% [14].

Neuroimaging played a crucial role in the case of this patient by depicting both the dural and leptomeningeal components of the disease. MR, including gadolinium-enhanced T1-weighted sequences, using at least 1.5T field strength, is considered the gold standard for the comprehensive neuroradiological assessment of patients with suspected leptomeningeal disease spread [11, 12].

Both types of lesions depicted avid contrast enhancement, reflecting their vascular nature and lack of a blood-brain barrier [6]. The extent of disease, in this case, has permitted its visualization in practically all MR sequences. Despite that, the contrast uptake made the smallest lesions even more conspicuous and let us learn the full extent of the disease, being essential in the characterization study of these patients [15]. It is crucial to image the entire spine from sacrum to foramen magnum in all patients with suspected malignant cord compression [16]. Contrast-enhanced computed tomography is less sensitive and may miss the diagnosis of leptomeningeal metastases in a third of cases [17].

The case we present had the complexity of both a compressive symptomatic dural mass and leptomeningeal disease being present at the same time which brought difficulties in the treatment. The approach, in this case, was to tackle the acute dorsal symptoms the patient was dealing with and radiation therapy was delivered to debulk the dural thoracic metastatic mass, with a moderate improvement in the symptoms after treatment. The role of RT in the palliation of bone metastases is well established. When compared to other primary solid tumors, patients with breast cancer tend to have a better response to radiotherapy when it comes to compressive symptoms and duration of remission [10].

The tumor board has regarded the possibility of intrathecal chemotherapy but the fact that the patient had a dural mass causing spinal cord compression made the debulking a priority. Intrathecal chemotherapy would mostly tackle the leptomeningeal component and not the dural mass [18]. Most series of patients treated for leptomeningeal metastases describe a global complication rate of 70%, including all grades of toxicity [12]. Since both treatments at the same time could carry an additional risk of toxicity, RT was preferred in order to reduce the compressive symptoms. In spite of that, several articles and guidelines [11, 12] advocate the use of both radiotherapy and chemotherapy in order to increase the possibility of reducing the tumor load.

## 4. Conclusion

Prolonged back pain with no apparent relief factor in a patient with a history of oncologic disease should alert the clinician to the possibility of metastatic spinal spread no matter how much time has passed since the initial diagnosis, and imaging should not be stalled in these cases.

MR with visualization of the entire spine is the examination of choice, and it will likely reveal or exclude the possibility of metastatic disease. The simultaneous presence of pachy and leptomeningeal metastases is uncommon and indicative of poor prognosis. The clear distinction between both is essential concerning patient orientation and treatment.
